# S. Giovanni Varieties (*Pyrus communis* L.): Antioxidant Properties and Phytochemical Characteristics

**DOI:** 10.1155/2019/6714103

**Published:** 2019-06-02

**Authors:** Elena Azzini, Giuseppe Maiani, Alessandra Durazzo, Maria Stella Foddai, Federica Intorre, Eugenia Venneria, Valentina Forte, Sabrina Lucchetti, Roberto Ambra, Gianni Pastore, Donato Domenico Silveri, Gianluca Maiani, Angela Polito

**Affiliations:** ^1^Council for Agricultural Research and Economics-Food and Nutrition, Via Ardeatina, 546 Roma, Italy; ^2^Department of Agriculture, Region of Abruzzo, Pescara, Italy

## Abstract

In order to evaluate and identify the antioxidant properties and the phytochemical characteristics, as well as the role of the genetic background and the different fruit parts in the definition of fruit quality, we characterized the existing germplasm through nuclear simple sequence repeats (SSRs) and evaluated the genetic divergence between ancient S. Giovanni varieties (*Pyrus communis* L.) and different types of grafting in relation to some variables of fruit quality. On the peel and pulp of pear varieties, the contents of flavanols, flavonols, and hydroxycinnamic acids as well as total antioxidant capacity were assessed. Their role in plant defences was confirmed by a significantly higher amount in the peel (206.66 ± 44.27, 48.45 ± 13.65, and 31.11 ± 11.94 mg/100 g, respectively) of S. Giovanni pears than in the pulp (71.45 ± 34.94, 1.62 ± 0.83, and 17.05 ± 5.04 mg/100 g, respectively). Data obtained using capillary analysis of SSR indicate unequivocally that the four samples of San Giovanni varieties can be divided into 3 different genetic groups. Cultivar and the different parts of the fruit can exert an action in the definition of the quality product. The use of local varieties and ecotypes can be considered a valid tool to improve food quality and at the same time to support local agrobiodiversity.

## 1. Introduction

The WHO [1] and guidelines for healthy Italian food habits recommend a daily intake of more than five portions (400 g) of fruits and vegetables. By the Passi National Report [2], in Italy, about 50% of adults consume no more than two servings of fruits and vegetables per day and less than 40% consume three to four servings, while only 1% consume the amount recommended by the guidelines for appropriate nutrition (five portions per day). Many researchers have shown that fruit and vegetable phytochemicals play a crucial role in the prevention of chronic diseases, also known as noncommunicable diseases (NCDs), including obesity, diabetes, cardiovascular diseases, and cancer [3–5], which represent an emerging global health issue. Hu D. et al. [6] have found an inverse association with pear consumption in a meta-analysis that includes evidences from prospective cohort studies about the association of fruits and vegetable consumption with the risk of stroke. The compounds of the greatest interest for their antioxidant and functional properties include phenolic compounds, unsaturated fatty acids, carotenoids, phytosterols, and tocopherols as well as flavonoids [7]. The pear, one of the oldest crops by humans [8], represents an important source of biologically active substances and is largely consumed worldwide. The aim of this research is to define antioxidant properties and phytochemical characteristics of S. Giovanni pear varieties. This is an autochthonous traditional variety of the Italian Abruzzo region with early ripening, not suitable for intensive cultivation, limited to local consumption, whose fruit trees are becoming rare.

## 2. Material and Methods

### 2.1. Pears

Four accessions including “Guastameroli,” “Casoli,” “Palmoli,” and “Civitella” of S. Giovanni varieties (*Pyrus communis* L.), from different locations, were propagated by grafting on the field of the native biodiversity of pear trees located at the Abruzzo region in the municipality of Scerni (Chieti). Pear accessions are grafted onto rootstock of the same species grown from seed (franco rootstock) or onto quince (quince rootstock), also obtained from seed. The field is located at the hilly company of the consortium for the development of techniques irrigated (Co.T.Ir.) owned by the Abruzzo region and covers an area of 1.3 ha, and the planting pattern adopted is 4 × 5 m (planted in rectangle with distances between the plants of 4 meters along the row and 5 meters between the rows). The place is on a gentle slope oriented to the south, the camp enjoys a good exposure, and there is a hill near a lake wherein the precipitation accumulates to be used for irrigation for the field itself by a drip system. The grafting assessed on a considerable amount (forty-six) of pear variety accessions showed a wide range of responses that can be summarized as follows: quince rootstock possessed poor affinity with pear cultivars, induced excessive reduction of tree size, and demonstrated signs of premature ageing trees in the presence of successful engraftment. All pears were collected at the optimum ripening stage recommended for consumption and delivered to the laboratory [9]. The pulp and peel were frozen in liquid nitrogen and crushed by a laboratory mill to a homogeneous powder in liquid nitrogen. Powders were kept in a refrigerator (-80°C) until extract preparation.

### 2.2. Materials

All solvents were purchased from Carlo Erba (Milan, Italy), BDH (Poole, England), and Merck (Darmstadt, Germany). 2,4,6-Tri(2-pyridyl)-s-triazine (TPTZ) was from Fluka (Switzerland). Phosphate-buffered saline (PBS), 6-hydroxy-2,5,7,8-tetramethylchroman-2-carboxylic acid (Trolox), and ascorbic acid were provided by Sigma-Aldrich Srl. Commercial standards were also from Sigma-Aldrich Srl (Milan, Italy). Double distilled water (Millipore, Milan, Italy) was used throughout the study.

### 2.3. Analytical Methods

*Total ascorbic acid* was extracted using Margolis and Shapira [10] by DTT (dithiothreitol) addition to reduce the dehydroascorbic acid. The quantitative analyses were performed by an HPLC system equipped with a coulometric detector (ESA model 580, Chelmsford, MA, USA). The setting potential was 0, 100, 200, 300, and 400 mV (v. Palladium reference electrode), and the chromatographic separation was obtained applying an isocratic elution at a flow rate of 0.8 ml/min [11].

*Extractable polyphenols* (EPP) were isolated according to Rufino et al. [12] with some modifications. Extractable polyphenols, which are readily solubilized by aqueous-organic solvents, comprise low molecular weight compounds from several classes and subclasses of polyphenols [13]. Further studies are needed and addressed for isolation of specific fractions of nonextractable compounds (NEPP), i.e., hydrolysable polyphenols (HPP), and nonextractable proanthocyanidins (NEPA) [14, 15]. Briefly, after weighing the sample, 20 ml of methanol/water (50 : 50 *v*/*v*, pH 2) solution was added to the samples. Samples were vortexed for 3-5 minutes and left under stirring for 1 h at room temperature in a water bath. After centrifuging the specimens at 2500 rpm for 10 minutes, the supernatant was recovered. 20 ml of acetone/water (70 : 30, *v*/*v*) solution was added to the residue for repeating the extraction, centrifugation, and recovery of the supernatant under the same conditions. Both methanol and acetone extracts were combined and centrifuged at 3500 g for 15 min. With respect to Rufino methodology, we have used an acid methanol/water (50 : 50 *v*/*v*, pH 2) as organic-aqueous solvent to improve the extraction efficiency. In addition, to better purify the extracts, a final step was added by centrifuging methanolic and acetonic extracts at 2800 g for 15 min. The resulting supernatant was transferred to falcon and directly used for the determination by colorimetric reaction with the Folin-Ciocalteau reagent [16].

The single compounds of the polyphenol fraction were extracted as described by Hertog et al. [17]. They consist of an extraction with methanol in the presence of the antioxidant BHT (butylated hydroxytoluene), followed by acidic hydrolysis with hydrochloric acid (HCl) 6 M at 90°C. The quantitative analysis through a system—ESA HPLC—with an electrochemical detector was reported by Azzini et al. [11].

Antioxidant properties were evaluated by FRAP (Ferric Reducing-Antioxidant Power) according to Benzie and Strain [18] and Pulido et al. [19]. This method represents a direct measure of the total reduction power of solution. The technique is based on the reduction of the complex between iron (III) and the tripyridyltriazine compound (TPTZ) by reducing compounds present in the food extracts which cause the formation of the iron (II)-TPTZ complex with the development of a blue color that can be monitored spectrophotometrically at a wavelength of 594 nm.

According to Re et al. [20], TEAC (Trolox Equivalent Antioxidant Capacity) measures the ability of antioxidants to scavenge the stable radical cation ABTS^+^ (2,2′-azino-bis(3-ethylbenzothiazoline-6-sulfonic acid). In the presence of antioxidants, a blue-green chromophore decreases in its intensity (maximum absorption at 734 nm).

#### 2.3.1. Genetic Authentication

A molecular approach to address food authentication and traceability using microsatellites or SSRs (simple sequence repeats) was performed. Based on a literature analysis [21], we have selected a statistically significant number of SSRs (ten) for the genetic characterization of pear samples, in order to investigate relationships between them. Genomic DNAs from pear samples (*n* = 4 for each sample) were extracted using the Sigma GenElute Plant Genomic DNA Miniprep kit following thoroughly the manufacturer's instructions. Samples were homogenized and pulverized under liquid nitrogen with a mini mill (IKA). All procedures included treatment with 0.3 *μ*g/*μ*l of RNase A and with 0.05 *μ*g/*μ*l of proteinase K. Primers amplifying pear SSR chosen for fingerprint analysis are reported in Table 1. DNA concentration and purity were determined using a NanoDrop 1000 spectrophotometer (Thermo Scientific). PCRs were performed in a total volume of 25 *μ*l containing 1x PCR buffer, 0.3 *μ*M of each primer, 2.5 mM MgCl_2_, 100 ng of DNA, 0.8 mM dNTPs, and 0.5 U Taq polymerase Gold (Applied Biosystems). The thermal protocol was as follows: an initial denaturation step at 95°C for 10 min followed by 28 cycles of 95°C for 30 s, 45 s at the appropriate annealing temperature, an elongation step at 72°C for 90 s, and finally, a step at 72°C for 45 min. Fragment size was extrapolated through capillary electrophoresis (Applied Biosystems 3730), including in the PCR reaction a specific labeled primer with 6-FAM (6-carboxyfluorescein).

### 2.4. Statistical Analysis

The results are presented as means with their standard deviation. Data analysis was performed using two-way analysis of variance (ANOVA) followed by the Bonferroni post hoc test (significance at *P* < 0.05). Pearson's linear correlation coefficient was used to evaluate the interactions between parameters. In addition, principal component analysis (PCA) was performed to determine the relationships between the pear cultivars to obtain an overview of correlation between pear quality trait as well as type of grafting.

## 3. Results

Plant phytochemicals play several and varied functions; their main activity is to protect plants from oxidative risk posed by various environmental stressors (sunlight and other environmental agents) and also to defend plants from fungal, bacterial, or viral infections. Plant phenolic content is composed of a heterogeneous mixture of molecules belonging to different families with varying chemical structures, and their content represents a peculiar characteristic of plant tissues [22].

As reported in Tables 2 and 3, three different classes of polyphenols were identified, namely, flavanols, flavonols, and hydroxycinnamic acids. Their role in plant defences was confirmed by a significantly higher amount in the peel (206.66 ± 4.27, 48.45 ± 13.65, and 31.11 ± 11.94 mg/100 g, respectively) of S. Giovanni pears than in the pulp (71.45 ± 24.94, 6.75 ± 3.04, and 17.05 ± 5.04 mg/100 g, respectively). In general, the flavanol content of the peel varied from 208.51 to 251.17 mg/100 g and 131.48 to 235.57 mg/100 g, respectively, for quince- and franco-type pears. In the pulp, flavanols ranged from 54.31 to 129.67 mg/100 g in quince fruits and from 33.91 to 82.83 mg/100 g in franco-type ones. The most representative flavanol in S. Giovanni pear varieties was proanthocyanidin B2, a dimeric form of epicatechin (epicatechin-(4*β*-8)-epicatechin).

Quince fruits “Civitella” possessed the highest proanthocyanidin B2 content in the peel (232.98 ± 3.72 mg/100 g). Also, the franco-type peel from Palmoli contained a relatively high amount of proanthocyanidin B2 (217.71 ± 11.86 mg/100 g), while its quince type showed the highest content in the pulp (120.12 ± 6.54 mg/100 g). Pears from “Casoli” showed a significant lower flavanol total content (*P* < 0.05) (131.48 ± 8.50 and 33.91 ± 4.43, respectively, for the peel and pulp) by comparison with other localities. The quince type showed a slightly higher amount of the total flavanol content (160.92 ± 78.89 mg/100 g) with respect to the franco type (124.48 ± 78.08 mg/100 g).

Total flavonoid content measured in the quince type (35.50 ± 28.8 mg/100 g f.w.) was higher too (*P* < 0.05) compared with the franco type (22.34 ± 17.84 mg/100 g). Quercetin-3-galactoside levels (20.93 ± 7.71 and 4.00 ± 0.13 mg/100 g in the peel and pulp, respectively) characterized the class of the flavonol content, also including quercetin-3-glucoside, quercetin-3-rhamnoside, and free quercetin. The flavonol total amount ranged from 61.31-64.33 mg/100 g to 3.93-7.68 mg/100 g of peel quince fruits and franco-type pulp, respectively.

As reported in Table 4, hydroxycinnamic acid content was represented mainly by chlorogenic acid, and its mean total average varied from 15.78 ± 5.02 and 29.11 ± 11.91 mg/100 g, respectively, for the pulp and peel. We found lower levels of p-coumaric acid in the pulp (1.26 ± 0.20 mg/100 g) and peel (1.41 ± 0.09 mg/100 g).

The content of EPP ranged from an average of 46.64 to 351.45 mg/100 g in the pulp and peel, respectively. The highest values were observed in the “Palmoli” pear (224.79 ± 131.81 and 195.96 ± 130.52 mg/100 g for quince- and franco-type pears, respectively), while the lowest values were observed in “Casoli” fruits (98.23 ± 53.71). The above standard deviations express and confirm the high variability in the distribution of these molecules in the several plant tissues.

Ascorbic acid content of S. Giovanni pears ranged from 8.19 to 23.29 mg/100 g in the pulp and from 17.74 to 46.84 mg/100 g in the peel. The lowest vitamin C content was measured in “Guastameroli” pears (12.96 ± 5.23 mg/100 g).

As shown in Table 4, there was a direct relationship between the total phenolic content and total antioxidant activity in phytochemical extracts of the peel and pulp. The peel of quince pears exhibited the highest FRAP that ranged from 12.56 to 14.07 mmol Fe++/100 g, respectively, for “Palmoli” and “Civitella” quince fruits (*P* < 0.05) comparing with the peel of Casoli and Palmoli franco fruits 6.46 to 11.34 mmol Fe^2+^/100 g, respectively. While no statistical differences were present between production areas, our results are well supported by the findings that extracts from peels showed significantly higher reducing power than the pulp ones. Similar trends were observed by scavenging of the ABTS^·+^ radical activities (TAC). Our findings showed significant differences (*P* < 0.05) comparing the peel and pulp on mean average of 5.30 ± 0.73 and 1.04 ± 0.73 mmol TE/100 g, respectively.

Table 5 displays the Pearson product moment correlation analysis between antioxidant activity and other phytochemicals, including different polyphenol fractions and vitamin C to investigate their relationship. From this analysis, a strong FRAP was positively correlated with the sum of flavonol phenolic fraction (*r* = 0.919). A lower relationship was assessed between FRAP and hydroxycinnamic acids (*r* = 0.728) and vitamin C (*r* = 0.628). The higher correlation was found between the EPP content and sum of flavonols (*r* = 0.981), too. The samples with a higher total phenolic content showed the highest antioxidant capacity (Table 5). We found that the highest antioxidant capacities were present in the “Civitella” quince type and the “Palmoli” franco type, respectively.

**Figure 1:**
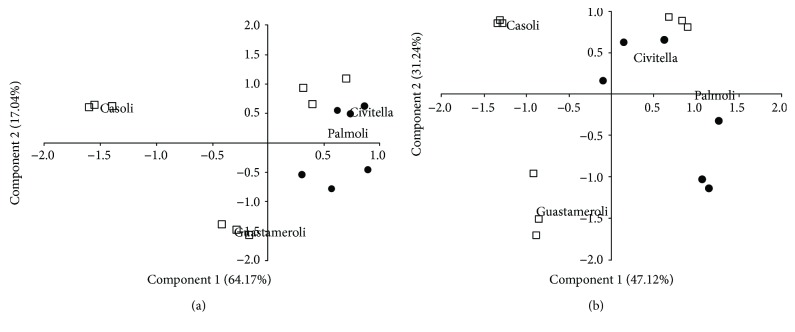
(a) Principal component analysis of the peel of the studied pear varieties. ☐: franco rootstock; •: quince rootstock. (b) Principal component analysis of the pulp of the studied pear varieties. ☐: franco rootstock; •: quince rootstock.

To assess the relationship between varieties, variables as measure of quality as well as type of grafting, the PCA was carried out and displayed for the peel and pulp separately. In our study, the PC1 and PC2 represented 81.21% and 78.36% of the system variance, respectively, for the peel (Figure 1(a)) and pulp (Figure 1(b)). In particular, for the peel, the first principal component explains 64.17% of the variance and the second 17.04%. In the pulp, the first and second components explain, respectively, the 47.12% and 31.24% of the total variance.

## 4. Discussion

The most representative flavanol in S. Giovanni pear varieties was proanthocyanidin B2, in agreement with the work of Ferreira D et al. [23], reporting that procyanidins were the main phenolics (96%) in a Portuguese pear variety. Also, Galvis-Sanchez et al. (2003) found that the flavonols are located largely in the peel respecting to the flesh, and the content of these compounds varied from 9.5 to 55.9 mg/100 g in the peel. Ozturk et al. [24] reported the chlorogenic acid as one major phenolic compound in the flesh and peel ranged from 1.58 to 89.12 mg/100 g and 2.10 to 134.84 mg/100 g, respectively. The chlorogenic acid level observed by Hudina et al. [25] ranged from 10.48 to 21.35 mg/100 g in the skin and 0.086 to 0.21 mg/100 g in flesh of the “Concorde” variety. Li X. et al. [26] reported that among the phenolic acids identified, the chlorogenic acid is the predominant (ranging from 3.25 to 44.33 mg/100 g) in the peels of ten pear cultivars, followed by p-coumaric acid (ranging from 1.41 to 16.48 mg/100 g). The authors reported a similar trend in the pulp, the chlorogenic acid ranging from 1.2 to 71.88 mg/100 g and coumaric acid ranging from 1.05 to 2.99 mg/100 g.

For the Spanish pear (*Pyrus communis* L. var Blanquilla), Gorinstein S. et al. [27] found a p-coumaric average of 5.17 ± 4.5 and 3.87 ± 0.31 mg/100 g f.w., respectively, for the peel and pulp. In addition, Ozturk et al. [28] detected p-coumaric acid as the minor hydroxycinnamic derivative. Its content varied from 0.020 to 0.164 mg/100 g in the flesh and from 0.030 to 0.169 mg/100 g in the peel of four European pear cultivars. These results were in line with previous findings that indicated a vitamin C content in the ranges from 9.1 to 29.7 mg/100 g in the flesh and 9.5 to 35.9 mg/100 g in the peel [29]. Moreover, vitamin C contents ranging from 11.6 to 22.8 mg/100 g in the peel and from 2.8 to 5.3 mg/100 g kg in the flesh were reported [29]. Ozturk et al. [30] reported a TP amount in the cultivars “Santa Maria” (43.8 mg/100 g) and “Deveci” (39.3 mg/100 g). Emerging evidence [31] suggests that phytochemical pear extracts are able to exhibit different levels of antimicrobial, antioxidant, and antimutagenic activities. In a systematic review on pear consumption and health outcomes, Reiland H and Slavin J [8] highlighted its healing properties. Overall, the peel of S. Giovanni varieties indicated a higher content of phenolic compounds than flesh, confirming the potential health benefit of the pear consumption as whole. Obviously, chemical fertilizer use should be taken into account, as it is known that they are able to cause a large number of negative health and environmental effects [32]. Moreover, the skin accounts for only 20% of the fruit, and while its intake does not affect the nutritional status, it could improve other human physiological functions through the presence of nonsoluble dietary fiber and vegetable waxes.

Several studies showed a negative association between dietary TAC and the incidence of degenerative diseases [33]; the TAC represents a suitable tool to evaluate the synergistic antioxidant properties of plant foods.

Our results were consistent with previous studies confirming the presence of a strong relationship between EPP and antioxidant activity [34]. Total hydroxycinnaminc acid content in the samples was also positively highly correlated with antioxidant activity measured as TEAC assay by radical cation (ABTS^·+^) (*r* = 0.661), which is in line with an earlier study that reported that antioxidant activity is closely related to the phenolic and flavonoid content.

Data obtained using capillary analysis of SSR PCRs indicate unequivocally that the four samples of San Giovanni varieties can be divided into three different genetic groups, as long as the “Palmoli” and “Civitella” showed identical genotypes. Data also indicate that the NB131a polymorphisms had lower discriminating power, as long as it was only able to identify the “Guastameroli” pears from all others (data not shown). DNA analysis of the accessions highlighted that there are three different species with a coincident ripening state. The relationship between pear varieties, some variables as measure of quality as well as the type of grafting, confirmed that (Figures 1(a) and 1(b)) the genetic background plays an important role in the definition of fruit quality as well as the type of grafting. These results represent a useful guide in selecting and breeding beneficial rootstocks for future genetic improvement programmes.

In summary, the consumption of these typical pear varieties due to its phytochemical composition could exert beneficial effects on human health, if its intake or processing occurs immediately after the harvest to minimize their losses.

## Figures and Tables

**Table 1:** Primers amplifying pear SSR chosen for fingerprint analysis.

Primer	Repeated motif	Forward (5′-3′)	Reverse (5′-3′)	bp
EMPc108	(CA)26	TGAGTGGGCTTTTGGTTTTC	TCCATTTAAACACATTTTCTGGA	122
NH002b	(GA)12	GGAGTCAGCGGCAAAAAAAG	CCCACTCCCTCCTCTTATTGT	180
NH029a	(AG)8	GAAGAAAACCAGAGCAGGGCA	CCTCCCGTCTCCCACCATATTAG	91-196
TXY11	(TC)8	CAGAATTCAACATTCACTCTCTCTC	GAGTAGGGATGTGTCGGCTC	120-166
TXY86	(AG)8	TTGGGTCTTTAAATGCCAGC	CCAGACGTGAGTTGTTGCC	114–156
EMPc01	(GT)17	AGTTTGGTATTGTGGAGGGTCTT	AGTCTTTTGGGTGGCTGAACA	135–197
EMPc11	(AC)13	GCGATTAAAGATCAATAAACCCATA	AAGCAGCTGGTTGGTGAAAT	121–161
EMPc110	(CT)18	ACTAACATTAAAAAATCTTTAC	ATCTTAAAACTTAAACTAAATAA	157–199
EMPc114	(AG)20	GTACCCACAATTCCCCATAT	GCCTTATGCGCCTTCTACC	152–169
NB131a	(GAA)4	GAGACCAAACAAAGCTGCCG	AACCCAACCCATCGAATCCC	261

**Table 2:** Sum and individual flavanol and flavonol contents in the peel and pulp (mg/100 g f.w.) of S. Giovanni pears from Abruzzo by varieties and type.

	Type	Flavanols				∑	Flavonols				∑
		Catechin	Epicatechin	B1	B2		QUE3GAL	QUE3GLU	QUE3RAMNO	QUE	
*Peel*											
Guastameroli (CH)	F	4.49 ± 0.10^cd^	12.90 ± 0.28^a^	0.66 ± 0.14^b^	188.53 ± 26.35^bc^	206.59 ± 26.71^d^	14.75 ± 0.48^bc^	11.34 ± 0.48^cd^	11.01 ± 0.52^ac^	1.54 ± 0.22^b^	38.64 ± 0.58^b^
Casoli (CH)	F	4.08 ± 0.13^bd^	5.51 ± 0.19^b^	2.39 ± 0.13^b^	119.50 ± 8.13^b^	131.48 ± 8.50^b^	11.58 ± 0.50^bc^	9.63 ± 0.56^d^	4.63 ± 0.54^b^	4.63 ± 0.54^a^	30.55 ± 1.17^c^
Palmoli (CH)	F	5.19 ± 0.38^ac^	11.16 ± 1.58^ac^	1.51 ± 0.04^c^	217.71 ± 11.86^a^	235.57 ± 13.8^ac^	19.77 ± 0.54^bd^	12.48 ± 0.57^c^	15.21 ± 3.09^a^	nd	47.46 ± 3.86^d^
Civitella (TE)	Q	5.70 ± 0.31^a^	10.30 ± 0.35^bc^	2.19 ± 0.23^c^	232.98 ± 3.72^a^	251.17 ± 3.50^a^	28.30 ± 2.26^a^	18.62 ± 1.52^b^	13.59 ± 1.84^ac^	0.80 ± 0.70^b^	61.31 ± 4.89^a^
Palmoli (CH)	Q	5.21 ± 0.95^ac^	11.51 ± 1.57^ac^	4.67 ± 0.35^a^	187.13 ± 3.35^bc^	208.51 ± 5.061^c^	30.22 ± 3.22^a^	22.51 ± 1.86^a^	9.40 ± 1.64^bc^	2.21 ± 0.15^b^	64.33 ± 3.37^a^
*ANOVA*		*P* < 0.05	*P* < 0.05	*P* < 0.05	*P* < 0.05	*P* < 0.05	*P* < 0.05	*P* < 0.05	*P* < 0.05	*P* < 0.05	*P* < 0.05
Total		4.94 ± 0.72	10.28 ± 2.75	2.28 ± 1.39	189.17 ± 41.94	206.66 ± 44.27	20.93 ± 7.71	14.92 ± 5.12	10.78 ± 4.07	2.30 ± 1.57^§^	48.45 ± 13.65
*Pulp*											
Guastameroli (CH)	F	3.81 ± 0.07	3.70 ± 0.31	nd^bd^	75.32 ± 14.23^bc^	82.83 ± 14.49^c^	3.93 ± 0.01^c^	nd	nd	nd	3.93 ± 0.01
Casoli (CH)	F	3.71 ± 0.04	3.41 ± 0.55	nd^bd^	26.79 ± 4.05^b^	33.91 ± 4.43^d^	3.86 ± 0.02^d^	1.91 ± 0.00	nd	nd	5.77 ± 0.01
Palmoli (CH)	F	4.03 ± 0.05	2.93 ± 0.11	0.65 ± 0.17^c^	48.90 ± 8.85^bc^	56.51 ± 9.17^cd^	4.11 ± 0.02^cd^	nd	3.57 ± 0.42^a^	nd	7.68 ± 0.43
Civitella (TE)	Q	4.16 ± 0.10	3.28 ± 0.35	0.64 ± 0.28^c^	46.30 ± 5.97^bc^	54.31 ± 6.81^cd^	4.08 ± 0.06^cd^	1.71 ± 0.59	6.82 ± 4.24^b^	nd	10.32 ± 5.47
Palmoli (CH)	Q	4.10 ± 0.21	4.21 ± 0.25	1.25 ± 0.00^a^	120.12 ± 6.54^a^	129.67 ± 4.55^a^	4.02 ± 0.24	2.01 ± 0.11	nd	nd	6.03 ± 0.35
*ANOVA*		ns	ns	*P* < 0.05	*P* < 0.05	*P* < 0.05	ns	ns	*P* < 0.05		ns
Total		3.96 ± 0.20	3.51 ± 0.53	0.85 ± 0.35^§^	63.47 ± 34.15	71.45 ± 34.94	4.00 ± 0.13	1.13 ± 0.3^§^	4.88 ± 2.78^§^	nd	6.75 ± 3.04
Fruit part^♦^		*P* < 0.001	*P* < 0.001	*P* < 0.001	*P* < 0.001	*P* < 0.001	*P* < 0.001	*P* < 0.001	*P* < 0.001	*P* < 0.001	*P* < 0.001

Data are shown as mean ± standard deviation of triplicate analysis. nd: not detectable. ANOVA by column: different letters indicate significant difference (*P* < 0.05); not significant (ns); ^§^mean of measurable compounds. ^♦^ANOVA: peel *vs*. pulp. F: pears from “franco” rootstock; Q: pears from quince rootstock.

**Table 3:** Sum and individual hydroxycinnamic acid contents (mg/100 g f.w.), extractable polyphenols (EPP) (mg/100 g f.w.), and vitamin C (mg/100 g f.w.) in the peel and pulp of S. Giovanni pears from Abruzzo by varieties and type.

	Type	Hydroxycinnamic acids			EPP (mg/100 g f.w.)	Vitamin C (mg/100 g f.w.)
		Chlorogenic (mg/100 g f.w.)	Coumaric (mg/100 g f.w.)	∑ phenolic acids (mg/100 g f.w.)		
*Peel*						
Guastameroli (CH)	F	25.83 ± 0.75^bc^	1.29 ± 0.01	27.12 ± 0.74^c^	194.42 ± 6.07^b^	17.74 ± 0.20^bc^
Casoli (CH)	F	11.61 ± 1.27^bc^	1.37 ± 0.04	12.98 ± 1.25^b^	147.24 ± 0.49^b^	37.03 ± 0.54^bd^
Palmoli (CH)	F	37.65 ± 5.64^ac^	1.43 ± 0.06	39.07 ± 5.70^a^	351.45 ± 42.55^a^	46.84 ± 4.21^a^
Civitella (TE)	Q	43.95 ± 6.02^a^	1.42 ± 0.07	45.37 ± 6.00^a^	245.74 ± 4.33^ab^	40.22 ± 0.72^bd^
Palmoli (CH)	Q	29.48 ± 3.06^a^	1.52 ± 0.06	31.00 ± 1.74^c^	314.56 ± 19.26^ab^	22.75 ± 0.47^bc^
*ANOVA*		*P* < 0.05	*ns*	*P* < 0.05	*P* < 0.05	*P* < 0.05
Total		29.11 ± 11.91	1.41 ± 0.09	31.11 ± 11.94	250.68 ± 79.66	32.91 ± 0.99
*Pulp*						
Guastameroli (CH)	F	12.46 ± 0.541^bd^	1.24 ± 0.01^ab^	13.70 ± 0.54^bc^	46.64 ± 3.89^a^	8.19 ± 0.07^bd^
Casoli (CH)	F	8.66 ± 0.38^bc^	1.22 ± 0.00^ab^	9.88 ± 0.38^b^	49.22 ± 2.18^a^	19.60 ± 3.94^ac^
Palmoli (CH)	F	16.23 ± 1.91^a^	1.46 ± 0.11^a^	17.69 ± 2.01^ac^	98.13 ± 18.33^b^	23.29 ± 0.66^ac^
Civitella (TE)	Q	21.04 ± 2.07^a^	1.06 ± 0.35^b^	22.09 ± 2.38^a^	72.55 ± 6.24^ab^	16.58 ± 0.05^bc^
Palmoli (CH)	Q	20.54 ± 0.20^bd^	1.33 ± 0.05^ab^	21.88 ± 0.15^a^	77.37 ± 4.87^ab^	17.53 ± 4.92^ac^
*ANOVA*		*P* < 0.05	*P* < 0.05	*P* < 0.05	*P* < 0.05	*P* < 0.05
Total		15.78 ± 5.02	1.26 ± 0.20	17.05 ± 5.04	68.78 ± 21.22	17.04 ± 5.69
Fruit part^♦^		*P* < 0.001	*P* = 0.001	*P* < 0.001	*P* < 0.001	*P* < 0.001

Data are shown as mean ± standard deviation of triplicate analysis. nd: not detectable. ANOVA by column: different letters indicate significant difference (*P* < 0.05); not significant (ns). ^♦^ANOVA: peel *vs*. pulp. F: pears from “franco” rootstock; Q: pears from quince rootstock.

**Table 4:** FRAP and TEAC in the peel and pulp of S. Giovanni pears from Abruzzo by varieties and rootstock type.

	Type	FRAP mmol Fe^2+^/kg	TEAC mmol Trolox/kg
*Peel*			
Guastameroli (CH)	F	9.42 ± 0.34	4.23 ± 1.63
Casoli (CH)	F	6.46 ± 0.3	3.38 ± 1.63
Palmoli (CH)	F	11.34 ± 0.81	4.02 ± 1.63
Civitella (TE)	Q	14.07 ± 0.82	4.65 ± 1.63
Palmoli (CH)	Q	12.56 ± 1.64	10.21 ± 1.63
*ANOVA*		ns	ns
Total		10.10 ± 1.16	5.30 ± 1.16
*Pulp*			
Guastameroli (CH)	F	1.04 ± 0.42	0.80 ± 0.41
Casoli (CH)	F	1.31 ± 0.16	0.57 ± 0.09
Palmoli (CH)	F	2.91 ± 0.07	1.35 ± 0.10
Civitella (TE)	Q	1.63 ± 0.14	0.63 ± 0.04
Palmoli (CH)	Q	2.34 ± 0.98	1.83 ± 0.95
*ANOVA*		ns	ns
Total		1.85 ± 0.82	1.04 ± 0.64

Data are shown as mean ± standard deviation of triplicate analysis. ANOVA by column not significant (ns). F: pears from “franco” rootstock; Q: pears from quince rootstock.

**Table 5:** Pearson correlation coefficients and *P* value between studied variables.

Variable	∑ flavanols	∑ flavonols	∑ hydroxycinnamic acids	Vitamin C	EPP	FRAP	TEAC
∑ flavanols		0.886 <0.001	0.836 <0.001	0.578 0.002	0.879 <0.001	0.864 <0.001	0.567 0.001
∑ flavonols	0.886 <0.001		0.785 <0.001	0.596 <0.001	0.918 <0.001	0.919 <0.001	0.661 <0.001
∑ hydroxycinnamic acids	0.836 <0.001	0.785 <0.001		0.552 0.001	0.801 <0.001	0.781 <0.001	0.411 0.03
Vitamin C	0.578 0.001	0.596 <0.001	0.552 0.002		0.694 <0.001	0.628 <0.001	0.254 0.175
EPP	0.879 <0.001	0.918 <0.001	0.801 <0.001	0.694 <0.001		0.874 <0.001	0.625 <0.001
FRAP	0.864 <0.001	0.919 <0.001	0.781 <0.001	0.628 <0.001	0.874 <0.001		0.436 0.02
TEAC	0.567 0.001	0.661 <0.001	0.411 0.03	0.254 0.175	0.625 <0.001	0.436 0.02	

## Data Availability

The data used to support the findings of this study are available from the corresponding author upon request.
